# Enhanced Sensitivity of a Resistive Pressure Sensor Based on a PEDOT:PSS Thin Film on PDMS with a Random-Height Micropyramid Structure

**DOI:** 10.3390/mi15091110

**Published:** 2024-08-31

**Authors:** Sungyong Kim, Dae Yu Kim

**Affiliations:** 1 Department of Electrical and Computer Engineering, College of Engineering, Inha University, Incheon 22212, Korea; ksy@inha.edu; 2 Center for Sensor Systems, Inha University, Incheon 22212, Republic of Korea; 3 Inha Research Institute for Aerospace Medicine, Inha University, Incheon 22212, Republic of Korea

**Keywords:** piezoresistive sensor, resistive pressure sensor, PDMS/PEDOT:PSS, micropyramid structure

## Abstract

The use of flexible pressure sensors has become increasingly widespread in a variety of applications, including wearable electronics and electronic skin. These sensors need to exhibit high sensitivity, wide detection limits, a fast response time, a linear response, and mechanical stability. In this study, we demonstrate a resistive pressure sensor based on randomly arranged micropyramid polydimethylsiloxane (PDMS) with a conductive poly(3,4-ethylenedioxythiophene): polystyrenesulfonate (PEDOT:PSS) thin film with a sensitivity of 391 kPa^−1^, a response time of 52.91 ms, a recovery time of 4.38 ms, and a limit of detection (LOD) of 0.35 kPa. Electrodes are then connected to a pair of the proposed resistive pressure sensors that face each other to fabricate a pressure sensing device. We examine various characteristics of the fabricated device, including the changes observed when applying loads ranging from 0 to 2.58 kPa. The proposed sensor exhibits high sensitivity and a rapid response time.

## 1. Introduction

Flexible pressure sensors are becoming increasingly common in wearable electronics [1,2], electronic skin [3,4], electronic devices [5], and robotics [6,7]. Pressure sensors measure pressure signals, which are defined as the ratio of a force to the area on which the force acts [8], and can be classified as piezoresistive [9,10], capacitive [11,12], or piezoelectric [13,14] depending on their sensing mechanism. Piezoresistive pressure sensors have received particular attention for a number of key advantages, such as their more straightforward manufacturing process, relatively high sensitivity, wide sensing range, and excellent anti-interference properties [15]. They can also be manufactured with a small size and consist of elastic conductive materials, allowing for a more flexible device structure and a wider deformation range [16].

Nevertheless, piezoresistive pressure sensors are expensive, cannot be mass-produced, and often exhibit poor stability [17,18]. In addition, there has been a drive to further increase the sensitivity of flexible pressure sensors so that they can be incorporated into sensing devices for the detection of other external signals, such as temperature and humidity [19,20]. To improve the sensitivity, it is important to tune the microstructure of the dielectric layer, which increases the manufacturing complexity of the sensor. As such, there remain a number of challenges to overcome to simplify this technology and increase its sensitivity.

The operational basis of piezoresistive pressure sensors is their ability to detect changes in resistance in response to external pressure stimuli, known as the piezoresistive effect. In this process, resistance *R* is expressed as

(1) $$R=\rho \frac{l}{s}$$

where *ρ* is the specific resistance of the material, *l* is its total length, and *s* is the cross-sectional area. The resistance of the material thus changes with l and s as it deforms.

Piezoresistive pressure sensors are typically fabricated with active materials positioned between two electrodes that are vertically aligned [21,22]. For decades, tremendous efforts have been made to improve sensor performance with bi- and trilayer microstructures [23,24], deformable electrodes [25,26], and novel durable materials [27]. The bi- and trilayer microstructures, consisting of flexible microstructured substrates as well as conductive electrodes, have been designed to enhance both stretchability and sensitivity. The deformable electrodes are typically made from conductive elastic composites with resistance changes in geometric dimensions or alterations in the local electrical percolation pathways. Novel durable materials based on textiles, cellulose, and biocarbon materials have been developed in applications of biocompatible and biodegradable pressure sensors [28]. Despite significant advancements in developing novel pressor sensors using artificial structures and conductive materials, the traditional approach has yet to fully overcome the trade-off between sensitivity and linearity in pressure sensors [29].

Electrode materials of flexible and stretchable electronics need to obtain high mechanical flexibility and optical transparency while maintaining electrical conductivity [30,31]. In response to these demands, researchers have concentrated on creating flexible functional materials to replace conventional rigid materials in electronic devices. Therefore, flexible electronic materials including metal wires or films, carbon nanomaterials, and conducting polymers have been investigated [32,33]. Among them, conductive polymers have recently received significant attention owing to their electrochemical and thermal stability, excellent electrical conductivity, and high transparency [34]. PEDOT:PSS is the most widely studied conducting polymer due to its high transparency in the visible spectrum, ease of solution processing, high work function, and significant mechanical stability [35]. However, since the pristine PEDOT film has a low conductivity of less than 1 S/cm, polar solvents of DMSO (dimethyl sulfoxide) and EG (ethylene glycol) are commonly used to enhance its electrical conductivity [36].

PDMS-based films with various surface and hierarchical microstructures have been developed for the use of pressure sensors [37]. The geometry and parameters of the microstructure have a significant influence on the contact area and local stress when pressure is applied. PDMS films with various microstructures including micropillar, micropyramid, and microdome arrays have been used to fabricate highly sensitive pressure sensors [38]. In general, pressure sensors with nano/microstructure patterns provide high sensitivity by significantly increasing the contact area under low-pressure conditions. However, as deformation progresses, the sensitivity of the pressure sensor greatly declines. Maintaining high sensitivity over a broad linear range is ideal for the best sensor performance [39,40]. An alternative approach to piezoresistive sensors is to produce PDMS microstructures with random pyramid architectures to enhance sensitivity by adjusting the height and spacing of the micropyramids [41]. Pressure sensors with irregular microstructures exhibit high sensitivity while maintaining broad linear sensing ranges [42,43]. When a small pressure is applied to the sensor, the contact area rapidly increases with the applied pressure due to the concentrated deformation of large microstructures, resulting in high sensitivity of the pressure sensor. As pressure increases, tiny microstructures contribute to electrical contacts, enabling pressure sensing over a wider linear range.

In the present study, we developed a low-cost pressure sensor with improved sensitivity and a fast response time by adding a PEDOT:PSS thin film to the surface of PDMS that had already been patterned with micropyramids of various heights. Cu-assisted chemical etching (CACE) was employed during the sensor fabrication process [44,45]. Etching times of 3, 5, and 10 min were also used with a silicon mold to produce micro-inverted pyramids with random heights. Previous microstructure-based pressure sensors were primarily manufactured using photolithography, 3D printing, and laser-assisted microengineering techniques [46,47]. However, these methods are often costly and time consuming. In contrast, the CACE process can create highly accurate inverted pyramid shapes on silicon surfaces. As the etching proceeds, the CACE selectively removes specific crystal planes, allowing for precise control over the three dimensions of the structure. This method enables the formation of either randomly arranged micropyramids or uniformly patterned structures with varying depths. Additionally, the CACE method uses inexpensive chemicals and does not require complex machinery to produce high-quality nanostructures [44,45]. The properties of the PEDOT:PSS composite were confirmed using Raman spectroscopy. Several peaks representing the specific vibrational modes of PEDOT and PSS were identified in the composite. A resistive pressure sensor based on the PDMS/PEDOT:PSS bilayer structure with micropyramids of random heights was developed using the CACE process and demonstrated improved sensitivity, a rapid response time, and high repeatability.

## 2. Experimental Section

### 2.1. Materials and Reagents

PDMS (Sylgard 184, silicon elastomer) and a corresponding curing agent were purchased from Dow Corning Co., Ltd (Midland, MI, USA). Cu(NO_3_)_2_, hydrogen peroxide (H_2_O_2_, 30%, GR), sulfuric acid (H_2_SO_4_), hydrogen fluoride (HF), toluene, and triclo(1H,1H,2H,2H-perflurooctyl)silane were purchased from Sigma Aldrich (Burlington, MA, USA). Si(100) wafers were purchased from Buysemi, Korea. All materials were used directly without further purification unless otherwise noted.

### 2.2. Silicon Mold Fabrication

The silicon mold was created using CACE. First, the boron-doped (1–10 Ω·cm), 500 μm thick, (100)-oriented, double-polished silicon wafers were thoroughly rinsed in acetone and deionized (DI) water for about 10 min to remove any organic contaminants. Wet etching of the silicon was then conducted using a Cu-based acid solution containing 5 mM Cu(NO_3_)_2_, 4.6 M HF, and 0.55 M H_2_O_2_ at 50 °C. The etching times were 3, 5, and 10 min. The residual Cu nanoparticles were subsequently removed using a concentrated nitric acid (HNO_3_) solution in a sonication bath.

### 2.3. PEDOT:PSS Composite

To prepare the PEDOT:PSS composite, a pristine PEDOT:PSS solution was stirred at 200 rpm and 40 °C to obtain the desired volume at a 2× concentration. Based on the weight of PEDOT:PSS, 10 wt.% of ethylene glycol (EG) and 1.5 wt.% of TX (Triton)-100 were added, and the solution was vortexed for 10 min at room temperature.

### 2.4. Pressure Sensor Fabrication

A piranha solution was prepared by slowly adding H_2_O_2_ to concentrated H_2_SO_4_ at a 3:1 ratio to control the exothermic reaction. The surface of the silicon mold was then soaked in the piranha solution for 30 min and rinsed with DI water. To facilitate the removal of the cured PDMS from the silicon mold without damaging the pattern, the surface of the silicon mold was coated with a self-assembling monolayer of trichloro(1H,1H,2H,2H-perfluorooctyl)silane (FOTS). The mold was placed in a sonication bath for 10 min and in a vacuum chamber for 60 min at room temperature. It was then annealed at 150 °C for 60 min. This process transformed the previously hydrophilic surface into a hydrophobic one.

The curing agent was mixed at a 10:1 ratio with the PDMS elastomer, and the mixture was cast onto the silicon mold. Oxygen (O_2_) and nitrogen (N_2_) contained in the mixture and residual air bubbles generated during mixing were extracted in a vacuum chamber to improve the quality of the mold by removing air bubbles inside the PDMS mixture. The PDMS was then cured at 70 °C for 4 h and peeled off the silicon mold. The pressure sensor was fabricated by casting the PEDOT:PSS composite onto the PDMS mold using spin coating (500 rpm for 20 s or 200 rpm for 60 s). The sensor was then annealed at 140 °C for 60 min.

### 2.5. Characterization and Measurements

The surface morphology of the silicon and PDMS molds was analyzed using a scanning electron microscope (SEM, Olympus OLS4500, Tokyo, Japan). Changes in the electrical resistance of the sensor due to changes in pressure were obtained using a source meter (Keithley 2400, Tektronix, Beaverton, OR, USA).

## 3. Results and Discussion

### 3.1. Preparation and Characterization

**Figure 1:**
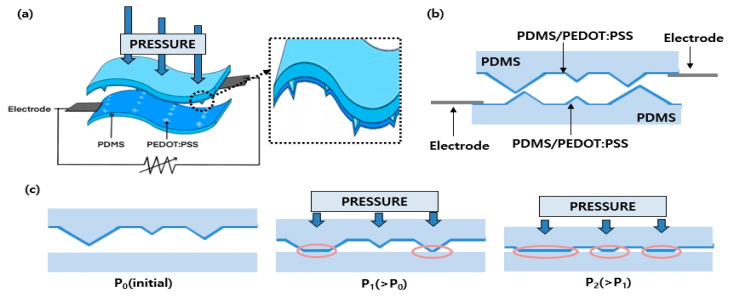
Operating principles for the proposed resistive pressure sensor based on a PEDOT:PSS thin film coating on microstructured PDMS with micropyramids of random height. (**a**) Mechanisms associated with the change in resistance when constant pressure is applied to the upper surface of the sensor. (**b**) Side view of a resistive pressure sensor based on a PEDOT:PSS thin film on PDMS with a micropyramid structure. (**c**) Morphological change in the sensor with a change in the sensor pressure.

Figure 1 summarizes the operating principles of the proposed resistive pressure sensor based on a PEDOT:PSS thin film coating on microstructured PDMS with micropyramids of random heights. The sensing mechanism is a change in resistance when a constant pressure is applied to the upper surface of the sensor. As shown in Figure 1a, when a certain pressure is applied to the upper surface of the sensor, the change in resistance is measured. Figure 1b shows a side view of a pair of resistive pressure sensors based on the PDMS/PEDOT:PSS facing each other with electrodes connected to the top and bottom sensors. The total resistance of the sensor is determined by the resistance of the PDMS/PEDOT:PSS layer and the electrode. However, because the resistance of the electrode is fixed, the sensor’s resistance signal is mainly determined by the PDMS/PEDOT:PSS layer, as illustrated in Equation (1).

Figure 1c shows the morphological changes in the sensor with a change in pressure from an initial pressure *P*_0_ to *P*_1_ and *P*_2_. The sensitivity of the sensor is defined as

(2) $$S=\frac{∆R/R_{0}}{∆P}$$

where *R* is the sensor’s output resistance, Δ*P* is the change in pressure, and *R*_0_ is the sensor’s initial resistance. The change in resistance due to the change in pressure is expressed as ΔR/R_0_.

**Figure 2:**
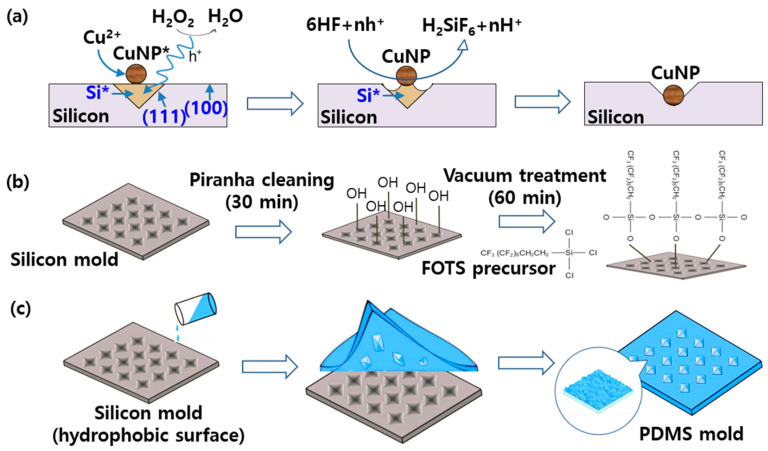
Fabrication process for the silicon and PDMS molds. (**a**) Time-dependent Cu-assisted chemical etching (CACE) to obtain an inverted pyramid structure. (**b**) Piranha cleaning process switches the surface of the silicon mold from hydrophobic to hydrophilic. (**c**) PDMS mold obtained from the hydrophobic silicon mold.

Figure 2 presents the fabrication process for the silicon and PDMS molds. Figure 2a shows the CACE process used to obtain an inverted pyramid structure via time-dependent etching. This selective etching is a proper method for creating pyramidal structures of random heights and has the advantage of processing large areas [44,45]. Copper nanoparticles (CuNPs) act as a catalyst to promote redox reactions on the silicon surface, and etching proceeds as HF removes the oxide film. First, copper ions (Cu^2+^) and hydrogen peroxide (H_2_O_2_) react on the silicon surface to generate Cu nanoparticles (CuNP*) expressed as follows:

Cu^2+^ + H_2_O_2_ → CuNP* (3)

In this process, a part of the silicon (Si) surface is oxidized to Si*, and H_2_O_2_ decomposes into H_2_O and O_2_. Next, the formed CuNPs promote etching of the silicon surface. Here, an etchant of HF (hydrofluoric acid) is used to selectively remove a specific crystal plane (111) of the silicon as follows:

Si* + 6HF + nh^+^ → H_2_SiF_6_ + nh^+^ (4)

Here, the silicon is continuously etched by repeating this process. In the early stage of etching, the process proceeds isotropically, but as time passes, the concentration of hydrogen peroxide decreases, and the etching proceeds anisotropically due to the influence of copper. CuNPs continue to promote etching, but silicon is etched only in a specific direction, forming the desired structure. After etching, residual copper is removed through concentrated nitric acid treatment, and the etched silicon surface forms an inverted pyramid structure according to the crystal direction. The etching times were 3, 5, and 10 min. After a predetermined etching time, the silicon substrate was removed from the etching solution and immediately immersed in water to prevent further etching.

As illustrated in Figure 2b, the piranha cleaning process makes the surface of the silicon mold hydrophilic via surface functionalization with silanol groups (Si-OH) during the oxidation process. For this reason, FOTS treatment was used to change the functionalized surface back to hydrophobic. Figure 2c shows the process of obtaining PDMS molds from the hydrophobic silicon molds.

**Figure 3:**
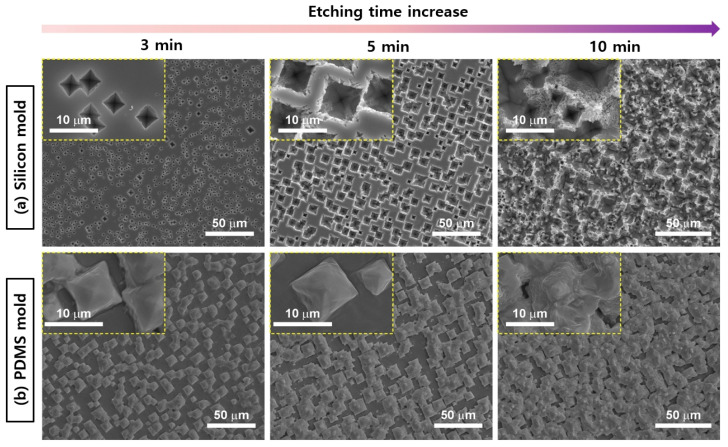
SEM images of silicon and PDMS molds. (**a**) SEM images of microstructures at a magnification of 50× on silicon molds etched for 3, 5, and 10 min in a mixture of 5 mM Cu(NO_3_)_2_, 4.6 M HF, and 0.55 M H_2_O_2_ (inset: higher magnification view of the microstructures). (**b**) SEM image of microstructures at a magnification of 50× on the PDMS mold fabricated from the silicon mold in (**a**) etched for 3, 5, and 10 min (inset: higher magnification view of the microstructures).

Figure 3 presents SEM images of silicon and PDMS molds. Figure 3a shows the surface morphology of a (100) silicon wafer etched using 5 mM Cu(NO_3_)_2_, 4.6 M HF, and 0.55 M H_2_O_2_, with the residual Cu nanoparticles removed. The etching rate for silicon wafers differs depending on the crystallographic orientation of the substrate, generally decreasing in the order of (100) ≈ (110) > (111). As the etching time increased from 3 to 5 to 10 min, an inverted pyramid structure was observed in the silicon mold. Etching for 3 min led to a uniform distribution of inverted pyramids over the surface, with the enlarged image revealing sharp, defined features. With 5 min of etching, the inverted pyramids were cleaner, deeper, and square-shaped, with the enlarged image showing sharper edges and a greater depth. After etching for 10 min, the surface clarity disappeared, and the enlarged image showed an irregular surface.

Figure 3b presents SEM images of the pyramid structure of the PDMS mold obtained from the silicon mold. With 3 min of etching, uniformly arranged pyramids appeared on the surface, with the enlarged image revealing distinct individual block shapes. After 5 min of etching, a uniform distribution of more pronounced, deep square-shaped pyramids was observed, with the enlarged image showing well-formed block shapes. With an etching time of 10 min, the surface pattern became irregular, and individual pyramid shapes became indistinct.

Collectively, the SEM images demonstrated that the etching time had a significant effect on the surface morphology of the silicon and PDMS molds. The silicon mold tended to exhibit more distinct and sharp features than the PDMS mold. Based on the results, 5 min was selected as the optimal etching time for both molds to obtain well-defined surface features, and these molds were used in subsequent analyses.

### 3.2. Experimental Results

**Figure 4:**
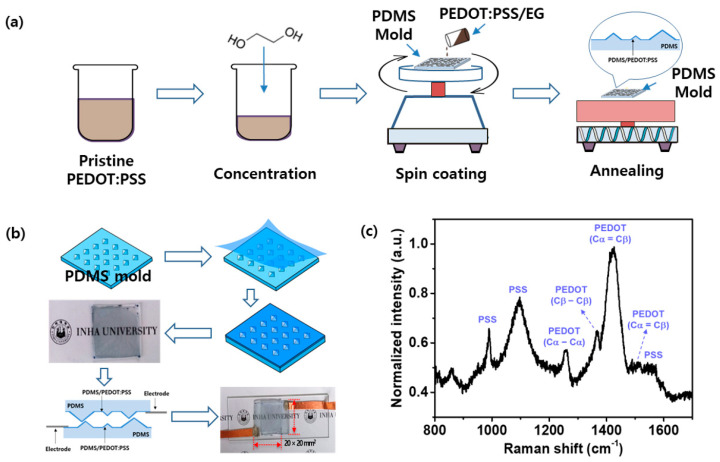
Fabrication process and Raman spectral characteristics of the PDMS/PEDOT:PSS-based resistive pressure sensor. (**a**) Coating of the PEDOT:PSS thin film on a PDMS mold. (**b**) Sensor device fabricated by attaching electrodes to PDMS/PEDOT:PSS sensors. (**c**) Raman scattering characteristics of the PEDOT:PSS composite.

The fabrication process and Raman spectral characteristics of the proposed resistive pressure sensor based on PEDOT:PSS thin films and PDMS with micropyramid structures of random height are presented in Figure 4. The PEDOT:PSS was coated onto the PDMS mold following the process outlined in Figure 4a, and Figure 4b displays the resulting resistive pressure sensor based on a PDMS/PEDOT:PSS, a pair of which are aligned to face each other and then connected to electrodes to produce the sensing device. The sensor was square, with a size of 20 × 20 mm^2^. Figure 4c presents the Raman scattering characteristics of the PEDOT:PSS composite. PEDOT is a conducting polymer, while PSS is nonconducting. Strong vibrations of the C*α* = Cβ bonds are the primary characteristic of the PEDOT peaks, whereas PSS peaks are more prominent at lower Raman shifts. In the Raman spectrum for PEDOT:PSS, the peaks at 1250 cm^−1^, 1365 cm^−1^, 1425 cm^−1^, and 1550 cm^−1^ are associated with vibrational modes of PEDOT, while the peaks at 990 cm^−1^ and 1180 cm^−1^ are characteristic of PSS. These distinct vibrational modes of PEDOT and PSS can be used to study the composition ratio, interactions, and structural changes within the composite.

**Figure 5:**
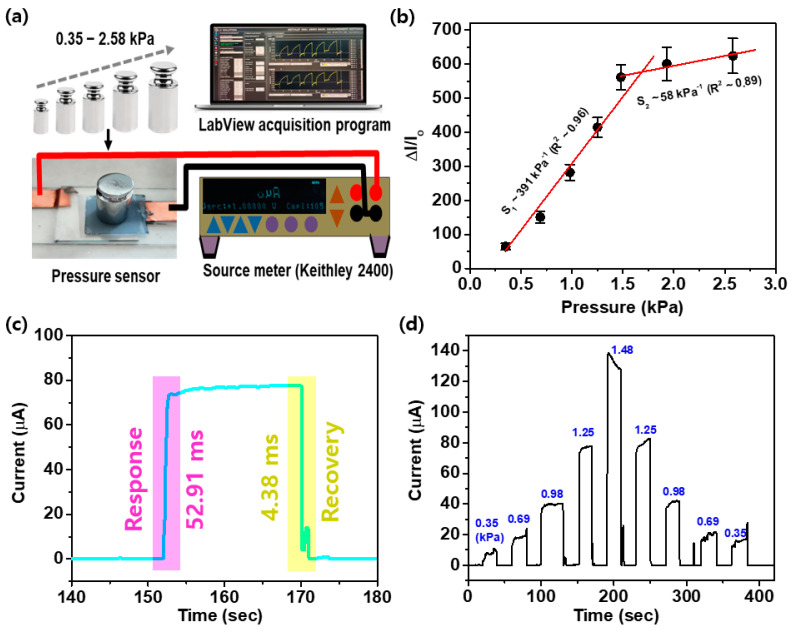
Pressure-sensing sensitivity and response characteristics of the proposed resistive pressure sensor based on PEDOT:PSS-coated PDMS with micropyramid structures of arbitrary heights. (**a**) Schematic of the pressure measurement setup. (**b**) Relative current changes (ΔI/I_0_) according to different pressures (kPa) showing the dynamic range and linear range of the developed pressure sensor. (**c**) Response and recovery time. (**d**) Current response to changes in the pressure. Each peak represents the sensor’s response to a change in pressure.

Figure 5 shows the pressure sensitivity and response characteristics of the resistive pressure sensor based on PDMS coated with PEDOT:PSS with micropyramid structures of arbitrary height. Figure 5a shows a schematic diagram of the pressure measurement setup. The measurements were performed by placing precision weights corresponding to pressures in the range of 0.35 kPa to 2.58 kPa on the sensor surface, and the data were obtained using a Keithley 2400 source meter operated by the LabVIEW acquisition program.

Figure 5b shows the relative current changes (ΔI/I_0_) when pressure loads are applied to the sensor. As the pressure increases, the relative current increases linearly from a measurement sensitivity of 391 kPa^−1^ to 1.48 kPa. The R² value is 0.96, indicating a strong linear relationship between the pressure and current changes. The dynamic range of the sensor is 0.35 kPa to 2.58 kPa. The linear range of the sensor output responds linearly to the input pressure (0.3 to 1.48 kPa).

Figure 5c presents the response speed of the sensor in terms of the time required for the current to reach specific thresholds of 90% of the rise time (the signal rises from 10% to 90%) and 10% of the fall time (the signal falls from 90% to 10%). When an external load was applied, a rise time of 52.91 ms was observed. After reaching a consistent current with the load applied, the sensor’s initial current was restored when the external load was removed. The observed fall time was 4.38 ms. These short rise and fall times demonstrate that the sensor can respond quickly.

Figure 5d displays the current response pattern according to changes in pressure for the sensor. The numbers in blue text represent the pressure in kPa that corresponds to the current response of each peak. The results highlight the repeatability of the sensor response when the load pressure is repeatedly increased and reduced from 0 to 1.48 kPa. As the pressure increased, the current also tended to increase. In practical sensor applications, sensitivity and response time are important metrics, and our proposed sensor demonstrates high sensitivity and a rapid response time.

Table 1 summarizes recent advances in pressure sensors based on typical performance parameters including materials, sensitivity, pressure ranges, and response time. The PDMS/PEDOT:PSS sensor developed in this study exhibits higher sensitivity (391 kPa^−1^) than the other materials, especially in the low-pressure range (0 to 2.65 kPa). The PDMS/PEDOT:PSS sensor has a response time of 52.91 ms, which is comparable to some of the other sensors but faster than the MXene- and carbon nanostructure-based sensors and slower than the CNT/PDMS and aligned Ni/PDMS sensors.

**Figure 6:**
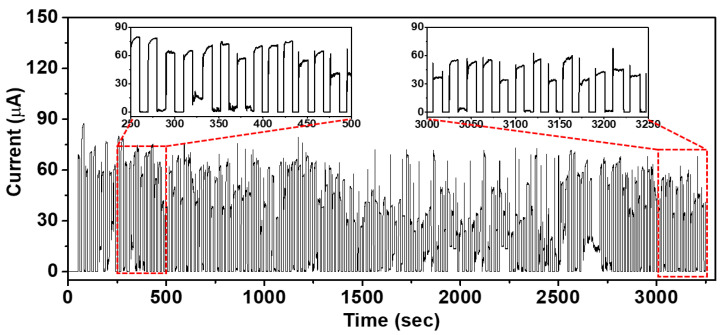
Current response of the proposed resistive pressure sensor based on microstructured PDMS/PEDOT:PSS when a pressure of 1.25 kPa is repeatedly loaded and unloaded for 150 cycles.

Figure 6 presents the cyclic loading/unloading test response characteristics of the resistive pressure sensor based on the developed PDMS with micropyramid structures of random height and a PEDOT:PSS coating. The response characteristics were measured using a source meter. The experiments used paired microstructured PDMS sensors (etching time = 5 min). To characterize the mechanical stability of the sensor, the device was continuously measured for approximately 150 cycles at a pressure of 1.25 kPa. Even after 150 cycles of measurement, the sensor maintained stable performance.

## 4. Conclusions

In this study, we developed and demonstrated a resistive pressure sensor with enhanced sensitivity based on a PDMS/PEDOT:PSS bilayer structure with micropyramids of random heights. The originality of this research lies in the fabrication of micropyramid structures with random heights through the CACE process as well as an evaluation of electrical characteristics for the use of highly sensitive pressure sensors. The sensing mechanism was the change in resistance to pressure when constant pressure was applied to the upper surface of the sensor. The resistive pressure sensor was fabricated using a silicon mold that was etched with etching times of 3, 5, and 10 min, and this mold was used to produce the PDMS mold. The structure of these molds was investigated using SEM images, and the properties of the PEDOT:PSS composite were analyzed using Raman spectroscopy. A sensor device with a pair of microstructured PDMS sensors (etching time of 5 min) was used to test the pressure-sensing sensitivity and response characteristics of the fabricated resistive pressure sensor device. The sensor characteristics were measured over a load range of 0 to 2.58 kPa. The sensitivity was 391 kPa^−1^ in the range of 0.3 to 1.48 kPa and the R² value was 0.96, indicating a strong linear relationship between the pressure and current changes. The dynamic range of the sensor was 0.35 kPa to 2.58 kPa. The sensor’s rise and fall times were 52.01 ms and 4.38 ms, respectively, representing a rapid response time. This newly developed resistive pressure sensor can be widely used in various applications including wearable electronic devices and electronic skin.

## Figures and Tables

**Table 1:** Several flexible piezoresistive pressure sensors reported in the literature.

Materials	Sensitivity	Pressure Range	Response Time	Reference
MXene	148.26 kPa^−1^	0~16 kPa	120 ms	[48]
Urchin-like hollow carbon spheres	260.3 kPa^−1^	1~10,000 Pa	30/60 ms	[49]
CNT/PDMS	15.1 kPa^−1^	-	4 ms	[50]
Carbon nanostructure on patterned PDMS	1.214 kPa^−1^	0~100 Pa	266/766 ms	[51]
Carbon nanofibers/PDMS	0.60 kPa^−1^	0~1 kPa	30/25 ms	[52]
MXene on patterned PDMS	2.6 kPa^−1^	0~30 kPa	40/40 ms	[53]
Aligned Ni/PDMS	0.72 kPa^−1^ at 357 kPa	373 kPa	~12/~20 ms	[54]
PDMS/PEDOT:PSS	391 kPa^−1^	0.3~1.48 kPa	52.91 ms	This work

## Data Availability

The data presented in this study are available on request from the corresponding author.
